# Persistent or new symptoms 1 year after a single high dose of vitamin D_3_ in patients with moderate to severe COVID-19

**DOI:** 10.3389/fnut.2022.979667

**Published:** 2022-09-13

**Authors:** Alan L. Fernandes, Lucas P. Sales, Mayara D. Santos, Valeria F. Caparbo, Igor H. Murai, Rosa M. R. Pereira

**Affiliations:** Rheumatology Division, Faculdade de Medicina HCFMUSP, Hospital das Clinicas, Universidade de São Paulo, São Paulo, Brazil

**Keywords:** post-viral stage, SARS-CoV-2, persistent symptoms, post-COVID-19, vitamin D, quality of life

## Abstract

**Purpose:**

The aim of this study was to investigate the reported persistent or new symptoms 1 year after a single dose of 200,000 IU of vitamin D_3_ and hospitalization in patients with moderate to severe COVID-19.

**Methods:**

This is a *post-hoc*, exploratory analysis from a multicenter, double-blind, placebo-controlled, randomized clinical trial from two hospitals in São Paulo, Brazil, registered in ClinicalTrials.gov, NCT04449718. Discharged patients were followed for up to 1 year and evaluated by telephone interviews at 6 and 12 months. The primary and secondary outcomes were previously published. These *post-hoc* exploratory secondary outcomes are the persistent or new symptoms and quality of life (QoL) at the post-viral stage of COVID-19. Generalized estimating equations (GEE) for repeated measures with Bonferroni’s adjustment were used for testing outcomes.

**Results:**

Between 2 June and 27 August 2020, we randomized 240 patients of which 144 were included in this study [the vitamin D_3_ (*n* = 71) or placebo (*n* = 73) group]. The mean (SD) age was 54.3 (13.1) years, and body mass index (BMI) was 32.4 (6.5) kg/m^2^. Fever demonstrated a significant main effect of time (*P* < 0.001) with a reduction from baseline to 6 (52–0) and 12 months (52–0). No significant differences between groups were observed for fever, cough, fatigue, fever, myalgia, joint pain, runny nose, nasal congestion, sore throat, hypertension, diabetes, cardiovascular disease, rheumatic disease, asthma, chronic obstructive pulmonary, chronic kidney disease, QoL, and new or persistent symptoms up to 1-year of follow-up.

**Conclusion:**

The findings do not support the use of 200,000 IU of vitamin D_3_ compared to placebo for the management of persistence or new symptoms, and QoL reported by moderate to severe patients after hospitalization for COVID-19.

## Introduction

Coronavirus disease 2019 (COVID-19) has been associated with manifestations in the post-viral stage that affect multiple organ systems (1). Emerging literature has reported residual effects of severe acute respiratory syndrome coronavirus 2 (SARS-CoV-2) infection manifested by a wide spectrum of symptoms that persist or begin after the acute-viral stage called “long-term” of COVID-19 (2–4), “long COVID” (5, 6), or “post-COVID-19 syndrome” (7, 8). According to the guideline (9), “acute COVID-19” represents signs and symptoms for up to 4 weeks, while “post-COVID-19 syndrome, long-term or long COVID” comprises signs and symptoms that develop during or after an infection consistent with COVID-19 present for more than 12 weeks and is not attributable to alternative diagnoses (4, 9).

Persistent symptoms in survivors of COVID-19 who had been hospitalized tend to vary according to the time of assessment. Carfi et al. (10) showed a high proportion of individuals who still reported fatigue (53.1%), dyspnea (43.4%), joint pain (27.3%), and chest pain (21.7%) 2 months after COVID-19 symptom onset. In a prospective cohort of patients interviewed by telephone after 4 months of hospital discharge by COVID-19, 51% reported the presence of at least one symptom that did not exist before the disease, being fatigue (31%), cognitive symptoms (21%), and new-onset dyspnea (16%) the most frequent manifestations (3).

Survivors of COVID-19 who had been hospitalized still present long-term health consequences at 6 months (2) or 1 year (11) after recovering from the acute phase of SARS-CoV-2 infection. Fatigue or muscle weakness (63%), sleep difficulties (26%), and anxiety or depression (23%) were the most common clinical manifestations after 6 months of symptom onset (2). A case report of a 50-year-old female nurse described a history of fatigue on minor exertion and persistent dysphonia without organic alterations after 1 year of mild COVID-19 (11).

Among numerous efforts to discover adjuvant therapeutic compounds against SARS-CoV-2 infection (12–14), it has been postulated through the effective increase in serum 25-hydroxyvitamin D (25[OH]D) levels after a single, safe high dose of vitamin D_3_ used in our study (15) the possible attenuate of the persistence or onset of symptoms. According to a previous systematic review (16), a single high dose of vitamin D_3_ significantly reduced levels of inflammatory cytokines (interleukin-6 and tumor necrosis factor-*alpha*, TNF-α) (17), improved endothelial function in patients with a history of stroke (18), type 2 diabetes (19), and cardiovascular function (20). Additionally, a single high dose of vitamin D3 promoted a good functional modified ranking scale with decreased mortality risk in ischemic stroke survivors with baseline serum 25(OH)D < 30 ng/ml at 6 months (21), while high concentrations of serum 25(OH)D were longitudinally associated with better quality of life (QoL) and less fatigue within 2 years after treatment (22).

Serum 25(OH)D levels are expected to remain sufficient for up to 4 months (23) following a single high dose of vitamin D_3_ of approximately 200,000 IU, even at baseline 25(OH)D levels 20 ≤ ng/ml (16). The main hypothesis was that a single high dose of vitamin D_3_ would sustain long-term serum 25(OH)D levels aiding in the modulation of inflammation, and innate and adaptative immune responses related to post-COVID-19 manifestation. In view of the biological plausibility and presumed benefit of vitamin D mitigating the persistent symptoms of COVID-19, this study investigates the reported persistent or new symptoms 1 year after a single high dose of vitamin D_3_ and hospitalization in patients with moderate to severe COVID-19.

## Materials and methods

### Study design

This is a *post-hoc* exploratory analysis of a multicenter, double-blind, placebo-controlled, randomized clinical trial (ClinicalTrials.gov, NCT04449718). The study and trial protocol were approved by the ethics committee and institutional review board of the Clinical Hospital of the School of Medicine of the University of São Paulo (Ethics Committee Approval Number 30959620.4.0000.0068) and were conducted in accordance with Good Clinical Practice guidelines and the Declaration of Helsinki. All patients provided written informed consent before being enrolled in the study and agreed to participate in the telephone interview at the post-viral stage of COVID-19. Complete information about the trial concept and design, data acquisition, analysis, and interpretation were previously published (15). The manuscript had been critically revised for important intellectual content and was approved for submission for publication by all the authors.

### Participants at the viral stage of COVID-19

Hospitalized patients were recruited from the Clinical Hospital of the School of Medicine of the University of São Paulo, and Ibirapuera Field Hospital from 2 June 2020 to 27 August 2020. The final follow-up occurred on 7 October 2020, and the screening criteria assumed were identical for both centers. All patients had a positive COVID-19 diagnosis confirmed by polymerase chain reaction (PCR) testing at the time of randomization or by serology assay (ELISA) to detect IgG against severe acute respiratory syndrome coronavirus 2 (SARS-CoV-2) due to the time from symptom onset to study enrollment.

Patients were eligible for enrollment if they were aged 18 years or older and had a positive SARS-CoV-2 infection diagnosis by either nasopharyngeal swab PCR or chest computed tomography scan with compatible findings (bilateral multifocal ground-glass opacities with at least 50% lung involvement). Patients were considered moderate to severe COVID-19 if they met the following hospital admission criteria: diagnosis of flu syndrome indicative of hospitalization, presenting with a respiratory rate greater than 24 breaths/minute, oxygen saturation lower than 93% on room air, or risk factors for complications (e.g., heart disease, diabetes, systemic arterial hypertension, neoplasms, immunosuppression, pulmonary tuberculosis, and obesity), followed by COVID-19 confirmation. Patients were excluded if they were unable to read and sign the written informed consent; they were already admitted under invasive mechanical ventilation; they had a recent previous vitamin D_3_ supplementation (greater than 1,000 IU/day or weekly equivalent); they had renal failure requiring dialysis or creatinine above 2.0 mg/dl; they had hypercalcemia defined by total calcium greater than 10.5 mg/dl; they were pregnant or lactating women; or if they were expecting hospital discharge in less than 24 h. The criteria used for hospital discharge were the absence of fever in the previous 72 h, no need for supplemental oxygen in the previous 48 h, and oxygen saturation greater than 93% on room air without respiratory distress.

### Randomization and masking at the viral stage of COVID-19

Eligible patients were assigned in a 1:1 ratio to either the vitamin D_3_ group or the placebo group. The randomization list was created using a computer-generated code, in bloc sizes of 20 participants, which was managed by a staff member who had no role in the study.

The vitamin D_3_ group received on the same day of randomization a single oral dose of 200,000 IU of vitamin D_3_ diluted in a 10-ml peanut oil solution. The selected dose is within the recommended range that appears to be most effective in promoting vitamin D sufficiency (16). Patients enrolled in the placebo group received only 10 ml of peanut oil solution. The vitamin D_3_ and placebo solutions were identical in color, taste, smell, consistency, and container. They were prepared by the pharmacy unit of the Clinical Hospital. Both were labeled by a staff member who did not participate in the study, and allocation blindness was maintained until the final statistical analysis.

### Procedures at the viral stage of COVID-19

Self-reported anthropometric characteristics (weight and height) and coexisting chronic diseases, acute COVID-19 symptoms, patients’ concomitant medications during hospitalization, oxygen supplementation requirement, and imaging features were assessed upon hospital admission. Self-reported coexisting chronic diseases and previous medications were checked according to the medical records for each patient. To provide a comprehensive demographic characterization, self-reported race/ethnicity data were also collected based on the following fixed categories: White, Black, Asian, and Pardo, the latter refers to people of mixed race/ethnicities, according to the Brazilian Institute of Geography and Statistics—IBGE (24).

Serum levels of 25-hydroxyvitamin D were assessed by a chemiluminescent immunoassay (ARCHITECT 25-OH Vitamin D 5P02; Abbott Diagnostics), at the same time by a blinded technician, following the manufacturer’s recommendations.

### Outcomes

The primary outcome, length of hospital stay, was previously published and did not differ between the vitamin D_3_ and placebo groups (15). The prespecified secondary outcomes were the number and severity of symptoms at the viral stage of COVID-19. To provide a comprehensive understanding of the effect of vitamin D_3_ in the post-viral stage of COVID-19, a secondary and exploratory *post-hoc* analysis was performed following reports from patients interviewed by telephone about persistent or new symptoms at 6 months and 1 year after hospital discharge and QoL at 6 months from hospital discharge. The QoL was assessed using the 36-Item Short Form Health Survey (SF-36), a generic questionnaire that was chosen due to concerns about the measurement properties of multiple domains and accuracy (25). The scale ranges from 0 to 100 for each of the eight domains and summary scores, and higher scores reflect better QoL. Investigators were blinded to patient-reported persistent symptoms and SF-36 responses. Score analysis was performed after completed assessments.

### Statistical analysis

The sample size was chosen based on feasibility and resources, as described in detail in a previous study (15). Generalized estimating equations (GEE) for repeated measures were used for testing possible differences in the persistence of symptoms and coexisting disease outcomes assuming group and time as fixed factors, with a binomial distribution, and a first-order autoregressive correlation matrix to test the main and interaction effects. Bonferroni’s adjustment was performed in GEE analyses to maintain a family-wise two-sided significance threshold of 0.05, considering 15 pairwise comparisons for all outcomes. Fever was handled by the McNemar test in comparisons within and between groups. Continuous variables were analyzed by an independent *t*-test. Percentages were analyzed by chi-square (χ^2^) or Fisher’s exact test. To consider potential confounders, GEE was handled by 3 models: unadjusted, adjusted by the length of hospital stay, and adjusted by the center (hospitals from which patients were recruited), using a per protocol approach. From 6 to 12 months, data were missing for 11.8% of patients (*n* = 9 in the placebo group and *n* = 8 in the vitamin D_3_ group) due to lack of contact during follow-up and were handled by GEE models, with no imputation for missing data.

Kaplan-Meier estimate curves for time (months) manifesting symptoms related to COVID-19 after hospital discharge were compared between vitamin D_3_ and placebo groups using the log-rank, Breslow, and Tarone-Ware, with cessation of the symptoms being right-censored in the analysis. To avoid bias regarding the different weights of the event in the survival curve analysis of each test, log-rank, Breslow, and Tarone-Ware were presented accordingly (26). Statistical analyses were performed using the IBM-SPSS software, version 20.0. The significance level was set at a two-sided *p*-value < 0.05.

## Results

Of the 1,240 patients assessed for eligibility, 240 underwent randomization during the acute phase of SARS-CoV-2 infection, with 120 assigned to each group. Of the 110 patients discharged in the vitamin D_3_ group, 21 were excluded due to the absence of a telephone number, 8 were excluded due to lack of contact, 8 withdrew consent, and 2 did not receive vitamin D_3_. Of the 112 patients discharged in the placebo group, 25 were excluded due to the absence of telephone numbers, 10 were excluded due to lack of contact, 3 died after hospital discharge, and 1 withdrew consent (Supplementary Figure 1). Overall, mean (SD) age was 54.3 (13.1) years, BMI was 32.4 (6.5) kg/m^2^, 77 (53.5%) were men, 82 (56.9%) patients were White, 43 (29.9%) were Pardo, 18 (12.5%) were Black, and 1 (0.7%) was Asian (Table 1).

**TABLE 1:** Baseline demographic and clinical characteristics^a^.

Characteristic	Vitamin D_3_ group (*n* = 71)	Placebo group (*n* = 73)
Age, years	55.2 ± 12.5	53.4 ± 13.8
Sex, *n* (%)		
Male	37 (52.1)	40 (54.8)
Female	34 (47.9)	33 (45.2)
Race or ethnicity, *n* (%)		
White	37 (52.1)	45 (61.6)
Pardo^b^	21 (29.6)	22 (30.1)
Black	12 (16.9)	6 (8.2)
Asian	1 (1.4)	0 (0)
Body-mass index, kg/m^2^^c^	32.8 ± 6.1	32.1 ± 6.9
Body-mass index category, *n* (%)^c^		
18.5–24.9 kg/m^2^	4 (6.2)	9 (12.9)
25.0–29.9 kg/m^2^	18 (27.7)	21 (30.0)
≥ 30 kg/m^2^	43 (66.2)	40 (57.1)
Time for length of hospital stay, days	6.0 (4.0–8.0)	7.0 (5.0–10.5)
Time from symptom onset to randomization, days	10.0 (7.0–12.0)	10.0 (8.0–14.0)
Time from symptom onset to hospital discharge, days	17.0 (13.0–20.0)	18.0 (15.5–23.5)
Time from symptom onset to 1st interview, days	218.0 (191.0–252.0)	221.0 (197.0–248.0)
Time from symptom onset to 2nd interview, days	398.0 (378.0–413.0)	393.0 (373.0–411.7)
25-hydroxyvitamin D, ng/mL	21.8 ± 10.7	21.2 ± 8.1
Severe 25-hydroxyvitamin D deficiency at randomization, *n* (%)	10 (14.1)	9 (12.3)
Dose of glucocorticoid at randomization, mg^d^	5.7 ± 12.5	4.1 ± 3.8
Concomitant medications, *n* (%)		
Anticoagulant	65 (91.5)	59 (80.8)
Antibiotic	60 (84.5)	65 (89.0)
Glucocorticoid	48 (67.6)	45 (61.6)
Antihypertensive	41 (57.7)	32 (43.8)
Proton pump inhibitor	30 (42.3)	30 (41.1)
Antiemetic	29 (40.8)	37 (50.7)
Analgesic^e^	28 (39.4)	36 (50.0)
Hypoglycemic	13 (18.3)	12 (16.4)
Hypolipidemic	6 (8.5)	11 (15.1)
Thyroid	9 (12.7)	9 (12.3)
Antiviral^f^	1 (1.4)	1 (1.4)
Oxygen supplementation, *n* (%)		
Oxygen therapy	58 (81.7)	59 (80.8)
Non-invasive ventilation	8 (11.3)	11 (15.1)
No oxygen therapy	5 (7.0)	3 (4.1)
Computed tomography findings, *n* (%)^g^		
Ground-glass opacities ≥50%	35 (54.7)	39 (61.9)
Ground-glass opacities < 50%	29 (45.3)	24 (38.1)

*^a^*Values are mean ± SD, median (IQR), or n (%). Continuous variables were analyzed by an independent t-test. Percentages were analyzed by chi-square or Fisher’s exact test. COVID-19, coronavirus disease 2019. *^b^*Pardo is the exact term used in Brazilian Portuguese, meaning “mixed ethnicity,” according to the Brazilian Institute of Geography and Statistics. *^c^*Body mass index data were missing for 6.3% of patients (n = 3 in the placebo group and n = 6 in the vitamin D_3_ group). SI conversion factors: To convert 25-hydroxyvitamin D to nmol/L, multiply values by 2.496. 25-hydroxyvitamin D deficiency (<10 ng/ml). No patients in the vitamin D_3_ group and 8 (10.9%) patients in the placebo group remained with severe 25-hydroxyvitamin D deficiency at hospital discharge, which precludes a comparison of long-term symptoms. *^d^* Glucocorticoid information was standardized in dexamethasone doses. *^e^*Analgesic data were missing for 0.7% of patients (n = 1 in the vitamin D_3_ group). *^f^* Included 1 patient from the vitamin D_3_ group and 1 patient from the placebo group receiving 75 mg of oseltamivir two times per day for 5 days. *^g^* Computed tomography finding data were missing for 11.8% of patients (n = 10 in the placebo group and n = 7 in the vitamin D_3_ group).

Fever demonstrated a significant main effect of time with a reduction from baseline to 6 months (52 to 0, *P* < 0.001) and from baseline to 12 months (52 to 0, *P* < 0.001) in the vitamin D_3_ group compared to the placebo group from baseline to 6 months (53 to 0, *P* < 0.001) and from baseline to 12 months (53 to 0, *P* < 0.001) (Table 2). No significant difference between the vitamin D_3_ and placebo groups for fever was observed at baseline (52 vs. 53, *P* = 1.00) (Table 2).

**TABLE 2:** Persistence of symptoms and coexisting diseases from baseline to 1 year after hospital discharge for COVID-19.

Outcomes	Vitamin D_3_ group (*n* = 71)			Placebo group (*n* = 73)			*P* ^a^	*P* ^b^	*P* ^c^
	Baseline	6 months	12 months	Baseline	6 months	12 months			
COVID-19 symptoms, *n* (%)									
Cough*	63 (88.7)	0 (0)	3 (4.2)	59 (80.8)	0 (0)	2 (2.7)	0.54	0.54	0.53
Fatigue*	60 (84.5)	23 (32.4)	30 (42.3)	63 (86.3)	28 (38.4)	28 (38.4)	0.46	0.46	0.47
Fever^#,&^	52 (73.2)	0 (0)	0 (0)	53 (72.6)	0 (0)	0 (0)	1.00	-	-
Myalgia*	45 (63.4)	15 (21.1)	27 (38.0)	48 (65.8)	16 (21.9)	17 (23.3)	0.08	0.08	0.08
Joint pain*	26 (36.6)	35 (49.3)	23 (32.4)	24 (32.9)	36 (49.3)	20 (27.4)	0.75	0.76	0.75
Runny nose*	21 (29.6)	6 (8.5)	4 (5.6)	31 (42.5)	11 (15.1)	7 (9.6)	0.97	0.95	0.97
Diarrhea*	23 (32.4)	7 (9.9)	1 (1.4)	33 (45.2)	6 (8.2)	2 (2.7)	0.37	0.37	0.37
Nasal congestion^d^*	23 (32.4)	8 (11.3)	2 (2.8)	34 (34.3)	8 (11.1)	(2.8)	0.98	0.98	0.98
Sore throat*	28 (39.4)	3 (4.2)	1 (1.4)	20 (27.4)	5 (6.8)	2 (2.7)	0.31	0.31	0.30
Coexisting diseases, *n* (%)									
Hypertension	38 (53.5)	42 (59.2)	42 (59.2)	33 (45.2)	31 (42.5)	31 (42.5)	0.13	0.31	0.28
Diabetes	27 (38.0)	24 (33.8)	24 (33.8)	16 (21.9)	18 (24.7)	18 (24.7)	0.17	0.39	0.38
Cardiovascular disease	7 (9.9)	8 (11.3)	8 (11.3)	8 (11.0)	5 (6.8)	5 (6.8)	0.24	0.41	0.20
Rheumatic disease^e^	9 (12.7)	10 (14.5)	10 (14.5)	7 (9.6)	7 (9.6)	7 (9.6)	0.36	0.37	0.37
Asthma	2 (2.8)	3 (4.2)	3 (4.2)	5 (6.8)	3 (4.1)	3 (4.1)	0.09	0.09	0.24
Chronic obstructive pulmonary disease	6 (8.5)	2 (2.8)	2 (2.8)	2 (2.7)	2 (2.7)	2 (2.7)	0.30	0.15	0.56
Chronic kidney disease*,**	1 (1.4)	3 (4.2)	3 (4.2)	0 (0)	1 (1.4)	1 (1.4)	0.05	0.05	0.16

Values are n (% within a group). COVID-19, coronavirus disease 2019. Data were analyzed by generalized estimating equations (GEE) with binomial distribution and identity link function with a first-order autoregressive correlation matrix. ^a^P-value represents group by time interaction unadjusted. ^b^P-value represents group by time interaction adjusted by the length of hospital stay. ^c^P-value represents group by time interaction adjusted by the center (hospitals from which patients were recruited). ^#^GEE unable to compute due to numerical problems (data were analyzed by McNemar test at baseline). ^&^Effect of time within-group compared to baseline by McNemar’s test. ^d^Nasal congestion data were missing for 0.7% of patients (n = 1 in the placebo group). ^e^Rheumatic disease data were missing for 1.4% of patients (n = 2 in the vitamin D_3_ group). *P < 0.05 for main effect of time. **P < 0.05 for main effect of group. From 6 to 12 months, data were missing for 11.8% of patients (n = 9 in the placebo group and n = 8 in the vitamin D_3_ group) due to lack of contact during follow-up and were handled by GEE models, with no imputation for missing data.

**FIGURE 1:**
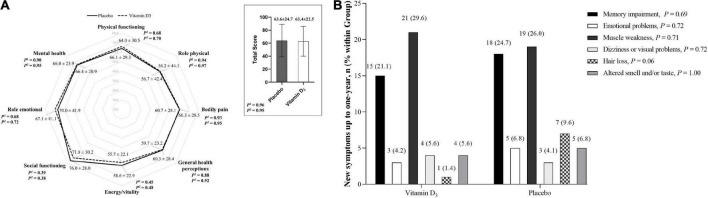
Quality of life and new symptoms related to COVID-19. **(A)** Quality of life was assessed using the 36-Item Short Form Health Survey (SF-36) at 6 months. Values are mean ± SD. Data were analyzed using an independent *T*-test and generalized estimating equations (GEE) with normal distribution and identity link function with the first-order autoregressive correlation matrix. ^1^*P*-value represents a 2-tailed independent *t*-test comparison. ^2^*P*-value represents the main effect of the group adjusted by the length of hospital stay. **(B)** Frequency of new symptoms from hospital discharge to 1 year of follow-up. Values are *n* (% within the group). Proportions were compared between groups using chi-square tests (χ^2^). New symptoms were missing for 11.8% of patients (*n* = 9 in the placebo group and *n* = 8 in the vitamin D_3_ group) due to a lack of contact during follow-up.

**FIGURE 2:**
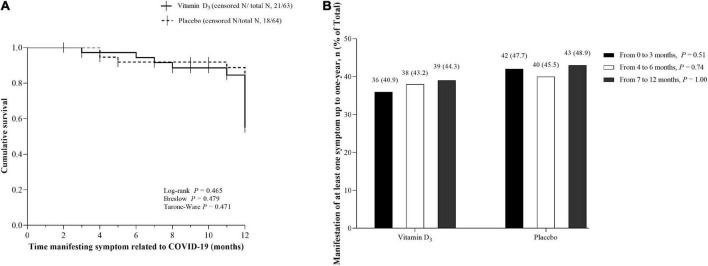
Kaplan-Meier curves for time manifesting symptoms and frequency of participants manifesting at least one symptom up to 1 year. **(A)** Cumulative survival for time (months) manifesting symptoms related to COVID-19 after hospital discharge in the vitamin D_3_ (42/63) and placebo groups (46/64). Vertical bars present single censored events (stop manifesting the symptom) in the vitamin D_3_ (*n* = 21) and placebo (*n* = 18) groups. **(B)** Frequency of participants manifesting at least one symptom from hospital discharge to 1-year follow-up. Values are *n* (% within the group). Proportions were compared between groups using chi-square tests (χ^2^). In panels A and B, data were missing for 11.8% of patients (*n* = 9 in the placebo group and *n* = 8 in the vitamin D_3_ group) due to lack of contact during follow-up, representing 127 patients in total.

No significant differences between the vitamin D_3_ and placebo groups for cough, fatigue, fever, myalgia, joint pain, runny nose, nasal congestion, sore throat, hypertension, diabetes, cardiovascular disease, rheumatic disease, asthma, chronic obstructive pulmonary, and chronic kidney disease were observed (Table 2). In addition, no significant differences between groups were found for QoL and frequency of new symptoms (Figures 1A,B), nor Kaplan-Meier curves for time manifesting symptoms (Figure 2A) and frequency of participants manifesting at least one symptom (Figure 2B) related to COVID-19 up to 1 year of follow-up.

## Discussion

In this *post-hoc* exploratory analysis from a multicenter, double-blind, placebo-controlled, randomized clinical trial, a single high dose of vitamin D_3_ did not significantly differ from a placebo for COVID-19 symptoms, coexisting diseases, or QoL. To the best of our knowledge, this is the first randomized clinical trial to investigate the reported persistent or new symptoms in patients with moderate to severe COVID-19 1 year after a single high dose of vitamin D_3_ and hospitalization.

Cough, fatigue, fever, myalgia, joint pain, runny nose, diarrhea, nasal congestion, and sore throat presented a significant main effect of time with reduced frequency from baseline to 6 and 12 months in both groups, except for the increase in chronic kidney disease, not differing between vitamin D_3_ and placebo.

The findings demonstrated manifestation of at least one symptom related to COVID-19 persisting for up to 1 year after the acute phase of SARS-CoV-2 infection in 44.3% of the participants in the vitamin D_3_ group and 48.9% in the placebo group. Furthermore, our results showed that from the first trimester after hospital discharge, at least 40% of participants did manifest any symptoms related to COVID-19 for both groups, in line with previous evidence (2, 3, 10), although we did not observe a significant difference in time to manifesting symptoms between the vitamin D_3_ and placebo groups.

The most frequent symptoms at 6 and 12 months were joint pain (49.3 and 32.4%, respectively), fatigue (32.4 and 42.3%), and myalgia (21.1 and 38.0%) in the vitamin D_3_ group compared to joint pain (49.3 and 27.4%), fatigue (38.4 and 38.4%), and myalgia (21.9 and 23.3%) in the placebo group. These findings are very similar to 53.1% fatigue and 27.3% joint pain at 2 months demonstrated by Carfi et al. (10), 31% fatigue at 4 months by the Comebac study (3), and 63% fatigue or muscle weakness at 6 months by Huang et al. (2).

Regarding new symptoms after hospital discharge for COVID-19, muscle weakness and memory impairment were among the most frequent in the vitamin D_3_ group, whereas in the placebo group, in addition to the two aforementioned symptoms, a trend (*P* = 0.06) of greater hair loss was observed in comparison to the vitamin D_3_ group, being the third most frequent manifestation.

The technical term for this hair loss is reported telogen effluvium. Telogen effluvium is characterized by diffuse hair loss within months of a significant systemic stressor, such as the premature follicular transition from the anagen (active growth phase) to the telogen (resting phase), the latter phase lasting approximately 3 months, after which excessive hair loss ensues (27). In fact, some evidence has pointed to SARS-CoV-2 infection as a precursor to acute telogen effluvium (28–32).

A research letter presented 10 patients with telogen effluvium following SARS-CoV-2 infection (28). All patients were women with a median age of 55, had laboratory-confirmed COVID-19, and had no history of hair loss. Mild symptoms were reported in six of them and severe disease in four, requiring hospitalization for an average of 7 days, and they all experienced excessive hair loss within months after infection, which included hair coming out in large clumps and thinning along the frontal hairline (28). These data corroborate other reports showing, on average, the onset of hair loss 50 days after the first symptom of COVID-19 infection (29, 31), associated or not with trichodynia (30), and may present duration ranging from 12 to 100 days (32).

One of the attributions to vitamin D functions would be related to hair loss. Vitamin D plays an important dermatological and dermatotherapeutic role in affecting the hair cycle due to its anti-inflammatory and immunomodulatory properties, and regulation of keratinocyte differentiation and proliferation (33). Recently, the benefit of oral vitamin D_3_ (200,000 IU) therapy in patients suffering from diffuse hair loss (telogen effluvium) has been demonstrated (34). However, conclusive studies regarding the presumed benefit of vitamin D in hair loss are lacking. There are no clinical trials that have investigated the efficacy of vitamin D in managing hair loss.

With respect to the QoL, Jacobs et al. (35) reported persistent symptoms at 35 days after hospital discharge for COVID-19 infection associated with lower odds of rating QoL and its categories (general health, physical health, mental health, and social functioning). Similarly, Chen et al. (25) demonstrated a poor health-related QoL among COVID-19 patients, and those women were negatively associated with physical function, bodily pain, and emotional domain at the 1-month follow-up. Even 4 months after hospitalization for COVID-19, the median SF-36 was 46.9 (IQR, 31.2–68.8) for vitality and 57.5 (IQR, 40.0–75.0) for general health (3). Our results show a slight, but not significant, improvement at 6 months, although we have failed to observe differences between the vitamin D_3_ and placebo groups.

During follow-up interviews, participants were also asked whether they had been using medications and/or supplements. Only 3 participants in the vitamin D_3_ group and 2 in the placebo group reported using a commercially available multivitamin or vitamin D (∼200 IU). Statistical sensitivity analysis excluding the aforementioned participants showed no significant difference in persistence or new symptoms, and QoL reported up to 1 year of follow-up between the vitamin D_3_ (*n* = 68) and placebo (*n* = 71) groups.

The postulated role of vitamin D in SARS-CoV-2 infection suggests action on the innate and adaptive immune system while mitigating excessive signaling for local and systemic inflammation (36). Our group failed to observe an effect of a single high dose of 200,000 IU of vitamin D_3_ eliciting significant changes in systemic inflammatory cytokines, chemokines, and growth factors compared with placebo (37). Furthermore, this supplementation strategy was not able to significantly reduce the length of hospital stay compared to those who were supplemented with a placebo (15). Nevertheless, clinicians and researchers continue the search for supplements with therapeutic properties that effectively mitigate post-COVID-19 syndrome (38).

Aside from experimental design, the strength of this study includes being a prospective trial investigating the effects of vitamin D_3_ on persisted or new symptoms after hospitalization for COVID-19, and enrollment of moderate to severe patients followed up for 1 year.

This study has several limitations. First, this investigation did not assess 25(OH)D concentrations at 6 and 12 months. Second, it was not feasible to evaluate the patients in person, and it was necessary to rely on the patients’ memory to obtain the data retrospectively. Third, regarding the new symptom onset related to COVID-19, it is not possible to determine when it started or ended. Fourth, at the time, most patients were admitted to the hospital’s emergency room, some of whom did not remember their telephone numbers, and the hospital’s social care staff did not locate the patients’ or family’s telephone numbers at hospital/study admission or during hospitalization; therefore, the authors are not able to state whether approximately 20% of participants excluded due to absence of telephone numbers would be alive after hospital discharge.

This study does not rule out the possibility that intermediate doses of vitamin D_3_ administered during follow-up could imply different results or that a single high dose of vitamin D_3_ followed by treatment with a recommended standard intake of at least 400 IU/day (39) may result in positive effects in favor of vitamin D and therefore could be evaluated in the future. Therefore, further studies are needed to prospectively assess the efficacy of fractionated high doses of vitamin D_3_ on persistent or new symptoms after hospitalization for COVID-19.

Despite the trend toward greater hair loss (telogen effluvium) as the third most frequent new symptom in the placebo group, we did not rule out the possibility that vitamin D could prevent marked telogen effluvium if applied as a booster dose around 3 months after hospital discharge. Our findings advance in showing that a single high dose of vitamin D_3_ did not support the initial hypothesis of an eventual role in innate and adaptive immune responses modulating long-term COVID-19, even in view of the expected significant reduction in COVID-19 symptoms from baseline up to 6 months and 1 year. Therefore, a single high dose of 200,000 IU of vitamin D_3_ compared to placebo did not interfere with the persistence or new symptoms, and QoL reported by moderate to severe patients after hospitalization for COVID-19 up to 6 months and 1 year of follow-up.

## Data availability statement

The raw data supporting the conclusions of this article will be made available by the authors, without undue reservation. Deidentified participant data of this study must be requested from the corresponding author RP, rosamariarp@yahoo.com. The codebook of this study will be made available upon request by qualified clinical researchers for specific purposes dependent on the nature of the request and the intention use of the data, with investigator support. The request must include a statistician. The lead author (RP) affirms that the manuscript is an honest, accurate, and transparent account of the study being reported; that no important aspects of the study have been omitted; and that any discrepancies from the study as originally planned (and, if relevant, registered) have been explained.

## Ethics statement

The studies involving human participants were reviewed and approved by the Ethics Committee of the Clinical Hospital of the School of Medicine of the University of São Paulo (approval numbers: 30959620.4.0000.0068). The patients/participants provided their written informed consent to participate in this study.

## Author contributions

RP: full access to all the data in the study and responsibility for the integrity of the data and the accuracy of the data analysis and funding and supervision. AF, IM, and RP: concept and design. AF and RP: drafting of the manuscript and statistical analysis. LS, MS, and VC: administrative, technical, or material support. All authors: acquisition, analysis, and interpretation, and critical revision of the manuscript for important intellectual content.

## Supplementary material

Supplementary Figure 1 Trial CONSORT diagram. All analyses were performed according to the patient’s randomization group using the intention-to-treat approach. There were missing data for 11.8% of patients (*n* = 9 in the placebo group and *n* = 8 in the vitamin D_3_ group) due to a lack of contact from the first interview at 6 months and the second interview at 1 year.

## Abbreviations

BMI: body mass index
COVID-19: coronavirus disease 2019
GEE: generalized estimating equations
IBGE: Brazilian Institute of Geography and Statistics
PCR: polymerase chain reaction
SARS-CoV-2: severe acute respiratory syndrome coronavirus 2
SF-36: 36-Item Short Form Health Survey
TNF-α: tumor necrosis factor *alpha*
25(OH)D: 25-hydroxyvitamin D.
