# Supplementary figures and images for: Persistent or new symptoms 1 year after a single high dose of vitamin D3 in patients with moderate to severe COVID-19
Source: Front Nutr. 2022 Sep 13;9:979667. doi: 10.3389/fnut.2022.979667 (PMC9513442; doi:10.3389/fnut.2022.979667)

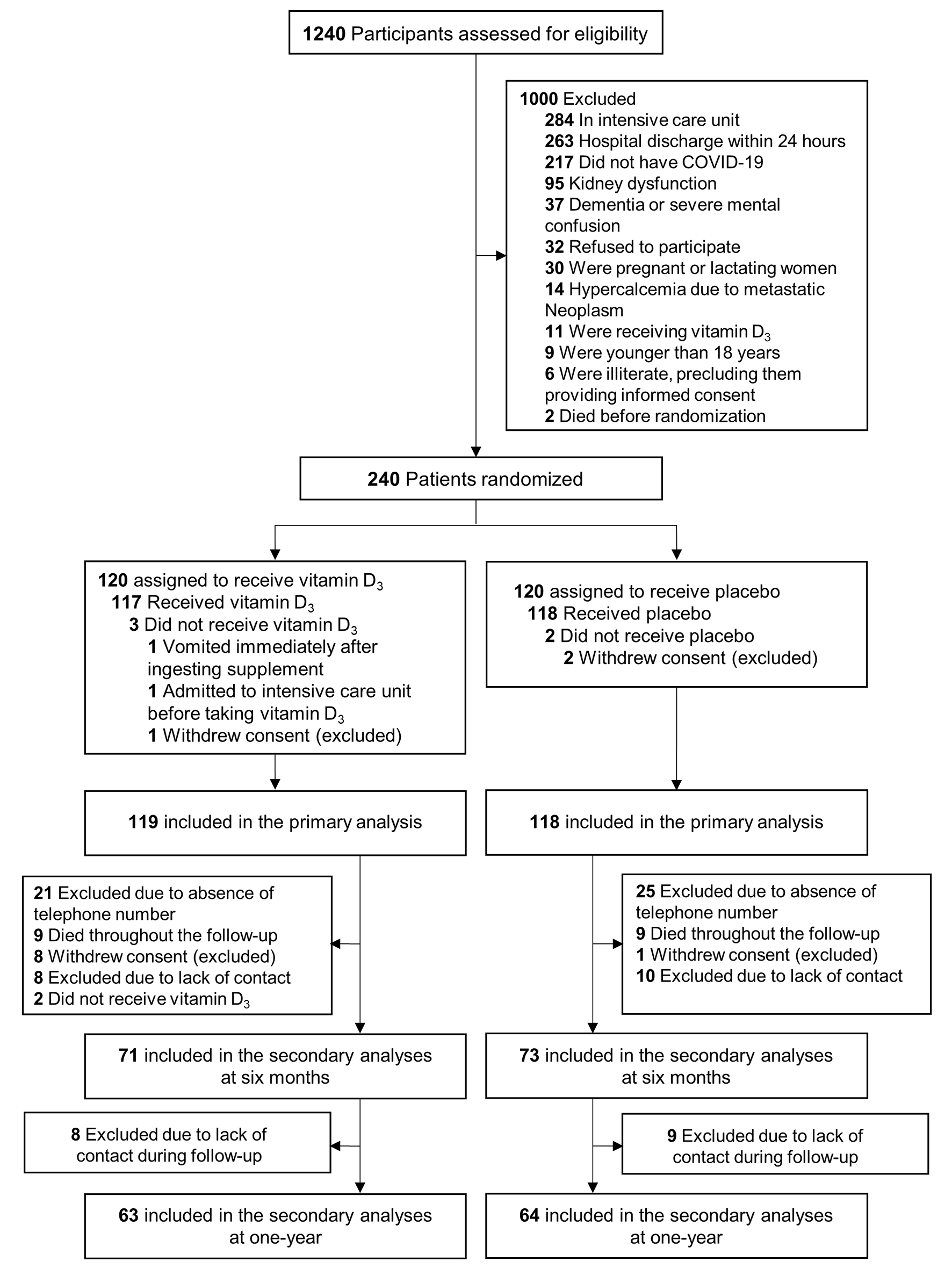

Supplement: Supplementary Figure 1 — Trial CONSORT diagram. All analyses were performed according to the patient’s randomization group using the intention-to-treat approach. There were missing data for 11.8% of patients (n = 9 in the placebo group and n = 8 in the vitamin D3 group) due to a lack of contact from the first interview at 6 months and the second interview at 1 year. [file Image_1.TIF]
